# A Woman with Chronic Subcutaneous Swelling of the Right Foot Associated with Sinus Tracts Discharging Yellow Grains

**DOI:** 10.1371/journal.pntd.0000772

**Published:** 2010-09-28

**Authors:** Ildefonso Tellez, Carlos Franco-Paredes

**Affiliations:** 1 Department of Medicine, Emory University School of Medicine, Atlanta, Georgia, United States of America; 2 Hospital Infantil de Mexico, Federico Gomez, Mexico City, Mexico; Weill Medical College of Cornell University, United States of America

## Case Description

**Figure 1:**
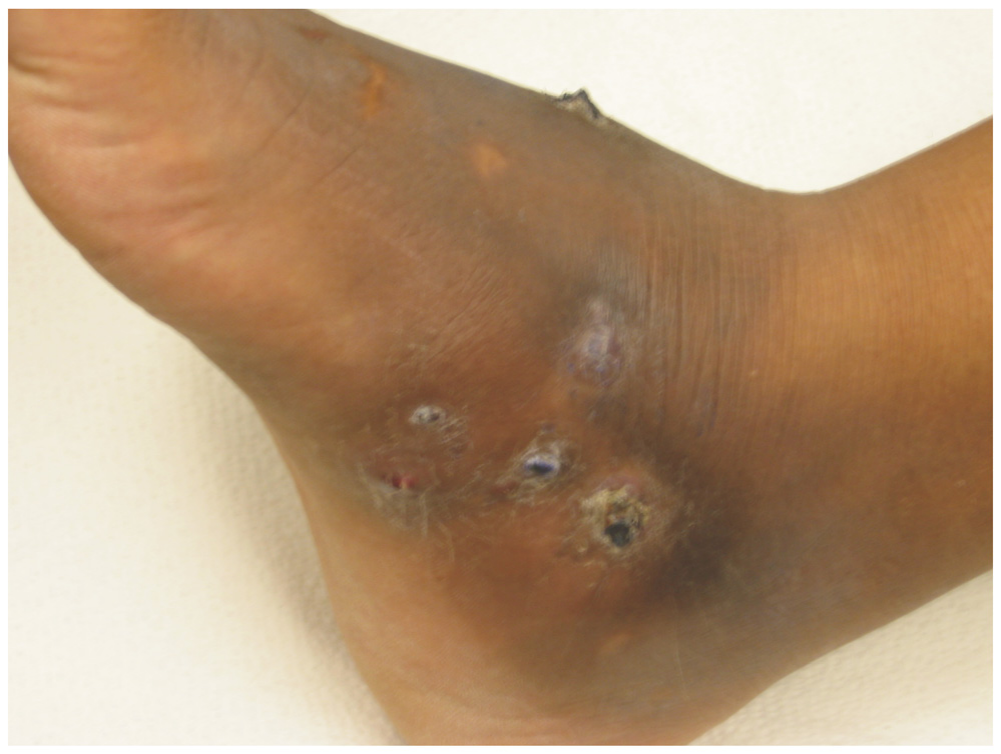
Photo of affected area.

A 41-year-old female from Mexico living in the United States over the past 7 years presented with a 4-month history of slowly progressive painless subcutaneous swelling and deformity on the anteromedial aspect of her right foot with multiple sinus tracts with intermittent serosanguineous drainage mixed with yellow grains (Figure 1). She reported the occurrence of a similar episode in the same affected area 13 years prior. At that point, she was treated with an unknown combination of two oral antibiotics for a 3-year period that resulted in significant clinical improvement.

Prior to coming to the US, she lived in an impoverished rural area of Guanajuato, Mexico. Since early childhood, she had participated in farming activities, and was often barefoot due to a lack of shoes. She denied any associated systemic symptoms such as fever, weight loss, or malaise. As the swelling progressively increased, she developed difficulty walking.

## Diagnosis

### 

#### Actinomycetoma caused by *Nocardia brasiliensis*

Mycetomas are chronic subcutaneous inflammatory granulomatous infectious processes that are divided into two types: eumycetoma or true mycetoma caused by fungi, and actinomycetoma caused by aerobic filamentous bacteria [1]–[6]. Actinomycetomas are caused by members of the genera *Nocardia*, *Streptomyces*, *Nocardiopsis*, and *Actinomadura*. Fungal mycetomas are most frequently caused by members of the genera *Madurella* and *Acemonium* [3]. Of these, *Madurella mycetomatis* is the most prevalent causative mycetoma-inducing agent [4], [5]. The term mycetoma, coined by Vandyke Carter in 1860, suggests a fungal tumor [1], ; however, aerobic filamentous bacteria cause most cases worldwide (60% of cases) [3], [5]. Mycetomas are also frequently identified as “Madura foot”. It is presumed that the first description of this entity occurred in 1842 in the Madras Medical Service of the British Army in India, and hence the term Madura foot [1], [7], [8].

Mycetomas occur among the poorest people living in resource-limited settings and produce considerable disability, disfigurement, and stigma. Mycetomas are therefore listed as neglected tropical diseases on the *PLoS Neglected Tropical Diseases* Web site (http://www.plosntds.org/static/scope.action) [9]. There is a wide geographical distribution of the various microorganisms that cause mycetomas depending on climate, rainfall, and ecologic factors [2], [3]. In general, mycetomas are endemic in relatively arid zones with short rainy seasons and low relative humidity [3]. A mycetoma belt has been described between the latitudes of 15°S and 30°N [1]. Mycetomas are usually endemic in some tropical and subtropical areas [2]–[5]. Most reported cases are from Mexico, Venezuela, Brazil, and Colombia in the Americas; India and Pakistan in the Indian subcontinent; and Somalia, Sudan, and Senegal in Africa [3]–[5]. Regardless of the causative microorganism, mycetomas tend to affect people who live in rural areas and are involved in outdoor activities [2], and onset of the microorganism establishment might result from traumatic inoculation of infectious fungi or bacteria present in the soil [3].

It has been suggested that eumycetomas usually predominate in Southeast Asia and Africa and actinomycetomas in the Americas. However, there is some data to suggest an increasing number of eumycetomas in South America [3]. *N. brasiliensis* is the most common cause of actinomycetomas, particularly in the Americas [3], [4]. In Mexico, mycetomas are often identified among impoverished farm workers in rural arid settings in the mainland states of Guanajuato, Oaxaca, and Morelos [2]. In these settings, actinomycetomas are responsible for 90% of mycetomas. *N. brasiliensis* causes 85% of cases, followed by *Actinomadura madurae* in 8% and *Streptomyces somaliensis* in 3% of cases. Eumycetomas are rarely reported in Mexico. The most frequent fungi have included *Madurella grisea*, *M. mycetomatis*, and *Acremonium* [2].

Clinical suspicion of mycetomas is based on the clinical triad that includes the presence of slowly progressive painless subcutaneous swelling, sinus tract formation, and granular discharge [1]–[5] affecting the foot in particular (80% of cases) as well as other parts of the body including the hand, head, neck, and back [2]–[4]. Actinomycetomas tend to progress more rapidly than eumycetomas, producing more inflammatory and destructive lesions [3]. In these sites, infection is usually localized, but involvement of deeper structures such as bone, tendons, and muscles may sometimes be identified [1], [4]. Dissemination to other organs such as the lung and vertebrae has been rarely reported [2], [3].

Diagnosis of mycetoma relies on direct examination of grains and isolation of the etiologic agents. The discharging grains represent aggregates of bacterial filaments or fungal hyphae [3]. The salient features of the grains may assist in the clinical diagnosis: eumycetomas due to *Madurella* spp. typically produce black grains [2]; actinomycetomas never produce dark grains, and usually are yellow to orange; and those caused by *Actinomadurae pelletieri* are red to pink [2], [4], [5]. Analysis of mycetoma sampled for further histological processing provides some clues to the potential microorganism, but culture is the gold standard for diagnosis [2], [3]. For fungi, molecular testing with PCR analysis has been developed since culture of some fungi may be challenging [3], [4]. Serologic testing has been shown to be useful in some settings, but in general it has demonstrated low sensitivity and specificity [1], [10]. Imaging studies are useful for defining the extent of disease; for example, ultrasound can be used to demonstratecharacteristic hyperreflective echoes and thick-walled cavities [1]. When available, computed tomography or magnetic resonance imaging or magnetic resonance imaging has been used to define the extent of disease.

Treatment of mycetoma is often challenging and depends mainly on the causative agent (bacterial or fungal) and severity of disease [1]–[3]. Actinomycetoma is amenable to medical therapy with prolonged courses of antimicrobials even in advanced cases [4], [5]. Combined drug therapy is always preferred to avoid drug resistance and to achieve microbiologic cure. The most common antimicrobials used are sulfas (cotrimoxazole and dapsone), aminoglycosides (streptomycin, amikacin), rifamycins (rifampin), tetracyclines (minocycline), beta-lactams (amoxicillin/clavulanate), or quinolones (ciprofloxacin) [3], [4]. Treatment of mycetoma caused by *N. brasiliensis* is less cumbersome than that caused by other bacteria [3]. A combination of dapsone and cotrimoxazole for 6 to 9 months is frequently used in resource-limited settings with good results. This combination is associated with substantial clinical improvement frequently observed within the initial 3 months of initiation of treatment [3]. More recently, the use of carbapenems (imipenem and meropenem) and oxazolidinones (linezolid) have shown to be effective in the therapeutic armamentarium against actinomycetomas, but their use remains limited in many settings due to cost and availability [2], [3], [6].

In contrast, for fungal mycetomas, a combination of medical and aggressive surgical approaches has been traditionally recommended [1]–[5]. Surgical debridement (sometimes requiring amputation) together with medical therapy (prolonged courses of triazoles) both prior to and after surgery is often the preferred therapeutic strategy [1], [5]. Recent data suggest that newer triazoles (voriconazole or posaconazole) may decrease the need of aggressive surgical interventions due to their higher efficacy, tissue penetration, and better bioavailability [2], [3]. However, their use needs tobe long-term, and thus, their broader use in resource-limited settings may be hampered by their cost [2].

In our patient, culture of one of the grains disclosed the presence of *N. brasiliensis*. Her current episode may represent a relapse of *N. brasiliensis* that was persisting from her initial episode, of 13 years ago [2], [4]. She has received a combination regimen of daily oral trimethoprim/sulfamethoxazole and oral dapsone for the past 3 months, with a reduction in foot swelling, and a decreasing number of sinus tracts and drainage.Our plan is to continue this antimicrobial combination for a prolonged period of time (6 to 12 months), depending on her clinical response to this regimen with laboratory monitoring (hemoglobin, methemoglobin levels, and basic metabolic profile) for any potential side effects.

**Key Learning Points**

- Mycetomas are divided into two types: eumycetomas caused by fungi and actinomycetomas caused by aerobic filamentous bacteria.
- Actinomycetomas generally respond more favorably to antimicrobial therapy compared to eumycetomas, which usually require surgical excision in addition to prolonged antifungal therapy.
- Combined drug therapy is always preferred for actinomycetomas with a combination of sulfa drugs (dapsone, bactrim) with either aminoglycosides (streptomycin, amikacin) and/or rifamycins (rifampin). Other alternative antimicrobials include tetracyclines, beta-lactams, or quinolones.
- Carbapenems and oxazolidinones have emerged as promising therapeutic strategies for actinomycetomas, and newer triazoles for the treatment of eumycetomas. However, their use in developing countries is currently limited by their availability and cost.
